# Supplementary figures and images for: Disruption of Marrow Microenvironments in Chronic Lymphocytic Leukemia by High-Resolution Synchrotron Micro-Computed Tomography
Source: bioRxiv. 2025 Oct 21:2025.10.20.683519. Preprint. [Version 1] doi: 10.1101/2025.10.20.683519 (PMC12633373; doi:10.1101/2025.10.20.683519)

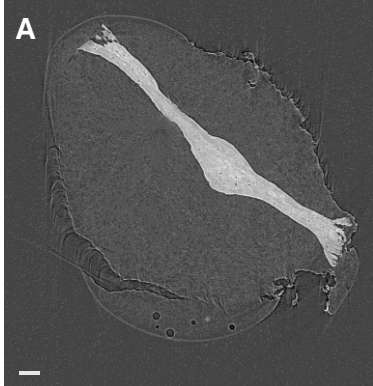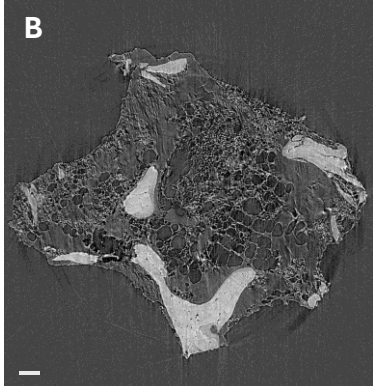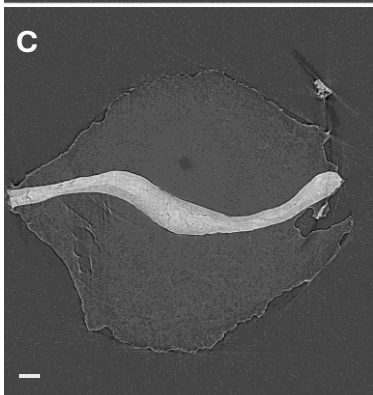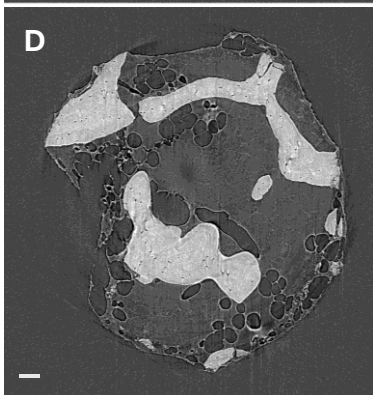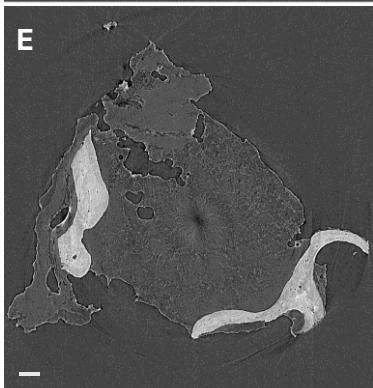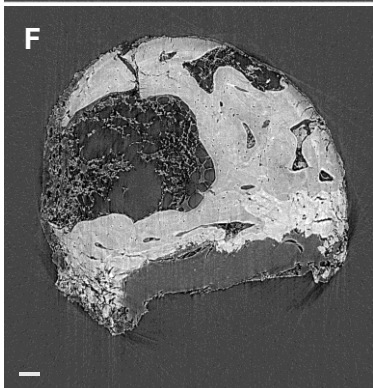

Supplement: Supplement 1 [file media-1.pdf]
